# Optimization of Bioactive Compound Extraction from *Prunus spinosa* L. Fruits Using Ultrasound-Assisted Extraction with Food-Grade Glycerin: A Combined RSM–ANN Approach

**DOI:** 10.3390/antiox15020202

**Published:** 2026-02-03

**Authors:** Asmaa Berkati, Nadir Ben Hamiche, Amina Kribeche, Louiza Himed, Salah Merniz, Maria D’Elia, Rita Celano, Luca Rastrelli

**Affiliations:** 1 Université de Bejaia, Faculté des Sciences de la Nature Et de la Vie, Laboratoire de Biomathématique, Biochimie, Biophysique et Scientométrie (L3BS), Bejaia 06000, Algeria; asma.berkati@univ-bejaia.dz (A.B.); nadir.benhamiche@univ-bejaia.dz (N.B.H.); amina.kribeche@univ-bejaia.dz (A.K.); 2 Department of Food Sciences, University of Mouloud Mammeri, Tizi-Ouzou 15000, Algeria; 3 Laboratory of Biotechnology and Food Quality (BIOCQUAL), Institute of Nutrition, Food and Agri-Food Technologies (INATAA), University of Constantine 1, Constantine 25000, Algeria; 4 Institute of Industrial Hygiene and Safety, University Batna 2, Batna 05000, Algeria; 5 Department of Pharmacy, University of Salerno, Via Giovanni Paolo II, 132, 84084 Salerno, Italy; 6 National Biodiversity Future Center (NBFC), 90133 Palermo, Italy; 7 Dipartimento di Scienze della Terra e del Mare, University of Palermo, 90123 Palermo, Italy

**Keywords:** blackthorn (*Prunus spinosa* L.), food-grade glycerin, ultrasound-assisted extraction, bioactive compounds, UHPLC-DAD-HRMS/MS, RSM–ANN modeling

## Abstract

Within the framework of green chemistry and wild fruit valorization, this study optimizes the extraction of bioactive compounds from *Prunus spinosa* L. fruits using glycerin-based ultrasound-assisted extraction (UAE). Response Surface Methodology (RSM) and Artificial Neural Networks (ANN) were comparatively employed to model the process. Significant improvements in extraction efficiency were achieved, with total phenolic content increasing from 9.28 to 23.22 mg GAE/g DW, total flavonoid content from 6.53 to 21.65 mg CE/g DW, and antioxidant activity (DPPH assay) from 57.04% to 86.34%. While both models performed well, ANN demonstrated slightly higher predictive accuracy, highlighting its potential for capturing complex, non-linear relationships in the extraction process. We identified the optimal extraction conditions as 9 min extraction time, 100% ultrasonic amplitude, and 40% water in glycerin, and these conditions were experimentally validated. UHPLC-DAD-HRMS/MS profiling revealed a rich phytochemical fingerprint dominated by phenolic acids, caffeoylquinic acid derivatives, and flavonol glycosides, and revealed largely overlapping qualitative phytochemical profiles between hydroglyceric and ethanolic extracts. Comparative extraction using 70% ethanol under identical conditions resulted in lower TPC, TFC, and antioxidant activity, indicating the improved efficiency of glycerin under the investigated conditions. Overall, the optimized glycerin-based UAE provides a sustainable, food-safe approach for extracting bioactive compounds from underutilized *P. spinosa* fruits. These results support its application in functional foods and in nutraceutical and cosmetic formulations.

## 1. Introduction

*Prunus spinosa* L., commonly known as blackthorn or sloe berry, is widely distributed across Europe and North Africa [1,2]. In Algeria, the species is locally referred to as “Barkouk lemaiz” in Arabic and “Lvarquq tɣeten” in the Kabyle Berber language. Blackthorn fruits are small, bluish-black spherical drupes with a greenish-yellow pulp, characterized by a distinctly sour and astringent flavor [3]. These fruits are particularly rich in bioactive polyphenolic compounds, including phenolic acids, flavonoids, and anthocyanins [4], which are associated with antioxidant, anti-inflammatory, and antibacterial properties, making them attractive for applications in the food, pharmaceutical, and cosmetic sectors [2]. Despite this growing interest, their efficient and sustainable recovery remains a key technological challenge.

In recent years, the food industry has increasingly focused on polyphenols due to their potential to support the development of eco-friendly products based on green raw materials, thereby reducing reliance on synthetic substances [5]. However, conventional extraction methodologies traditionally rely on organic solvents that present environmental and health concerns, including acute and chronic toxicity and potential carcinogenic effects [6]. This has encouraged the search for safer and more sustainable extraction alternatives. In this context, green extraction technologies have gained significant attention. These approaches aim to minimize or eliminate the use of hazardous solvents while maximizing the selective recovery of valuable bioactive compounds [7]. Among emerging green extraction techniques, ultrasound-assisted extraction (UAE) represents a sustainable and efficient strategy for the isolation of bioactive compounds. UAE enhances mass transfer through acoustic cavitation, allowing faster extraction, reduced solvent consumption, and improved reproducibility compared to conventional techniques. Importantly, UAE can be applied using a wide range of solvents, provided that solvent polarity and affinity toward target compounds are carefully considered. In this regard, glycerol has attracted increasing interest as a green solvent. Widely used in food, cosmetic, and pharmaceutical formulations, glycerol has demonstrated high efficiency in recovering polyphenols from plant matrices, while ensuring safety, stability, and sustainability of the resulting extracts [8].

The efficient recovery of polyphenols and flavonoids depends on multiple interrelated extraction parameters, whose individual and combined effects cannot be reliably evaluated using conventional one-factor-at-a-time approaches. Given the multivariate and interactive nature of ultrasound-assisted extraction processes, the use of Design of Experiments (DoE), such as Response Surface Methodology (RSM), represents a powerful strategy to systematically assess the combined effects of operating parameters while minimizing experimental effort. This approach provides a statistically robust framework for studying multiple factors simultaneously and exploring their interactions using a reduced number of experiments [9].

Among DoE-based strategies, RSM has been widely applied to predict optimal extraction conditions and to evaluate the combined effects of multiple variables on extraction efficiency [10,11]. This approach enables the visualization of factor interactions and the generation of predictive polynomial models, and has been successfully employed in ultrasound-assisted and microwave-assisted extraction processes [12,13]. While RSM offers clear interpretability of parameter interactions, it may show limitations when dealing with highly complex or non-linear systems, highlighting the potential benefit of complementary modeling approaches in specific extraction scenarios [12,13].

Artificial Neural Networks (ANNs) represent an advanced data-driven modeling approach inspired by biological learning mechanisms. By processing information through interconnected neurons and weighted connections, ANNs are capable of learning from experimental data and capturing complex, non-linear relationships between input variables and system responses [14]. Owing to their strong predictive performance, ANNs have been increasingly applied in food processing and extraction optimization studies, particularly in systems characterized by multiple interacting factors [12,15,16,17]. However, their applicability remains highly dependent on case-specific validation and the quality and size of the experimental dataset. In this context, ANN was employed as a complementary modeling approach to RSM in order to explore its ability to describe non-linear relationships within the same experimental design, rather than as a substitute for statistically interpretable regression models. Given the limited size of the experimental dataset, ANN results are discussed within the investigated design space and are not intended for broad extrapolation. Beyond extraction yield optimization, the integration of advanced analytical tools such as UHPLC-MS/MS is essential to characterize the phytochemical fingerprint of food-grade extracts and to assess solvent-driven selectivity toward bioactive compounds. Despite the continued popularity of RSM for modeling and optimization of extraction techniques, including ultrasound- [13] and microwave-assisted processes [11]. Systematic and direct comparisons between RSM and ANN applied to the same extraction system remain limited in the literature. To the best of the authors’ knowledge, no study has directly compared RSM and ANN for the extraction of bioactive compounds from *Prunus spinosa* fruits. However, comparative RSM–ANN approaches have been reported for other plant matrices, such as *Prunus nepalensis* [18] and *Sargassum horneri* [19]. These studies indicate that RSM and ANN exhibit complementary strengths, with RSM providing greater model interpretability and ANN offering enhanced flexibility in capturing non-linear relationships, thereby offering a relevant methodological framework for the present work. Despite the extensive application of RSM in green extraction studies and the growing interest in ANN-based modeling, a clear methodological gap still exists in the literature regarding the systematic and direct comparison of these two approaches when applied to the same ultrasound-assisted extraction system, particularly using food-grade green solvents and underexplored plant matrices such as *Prunus spinosa* fruits. Therefore, the objectives of this study were to: (i) optimize the extraction of bioactive compounds from blackthorn (*Prunus spinosa* L.) fruits using glycerin as a natural, food-grade solvent coupled with ultrasound-assisted extraction; (ii) investigate the effects of ultrasound amplitude, extraction time, and water concentration in glycerin on total phenolic content (TPC), total flavonoid content (TFC), and antioxidant activity (DPPH assay); (iii) validate the optimized extraction conditions and assess the effectiveness of glycerin compared to conventional 70% ethanol extraction; and (iv) compare the predictive performance of RSM and ANN models to identify the most suitable approach for describing and optimizing the extraction process.

## 2. Materials and Methods

### 2.1. Fruit Samples

Ripe fruits of *Prunus spinosa* L. (blackthorn) were manually harvested in September 2023 from wild plants growing in Minar Zareza, Mila region, Eastern Algeria, (geographical coordinates: 36°31′33.7″ N 5°52′38.4″ E). After harvesting, fruits were carefully sorted to remove damaged or defective samples and washed with distilled water to eliminate surface impurities. The cleaned fruits were air-dried on perforated racks at room temperature for 20 days until a pliable texture and reduced moisture content were achieved. Subsequently, seeds were manually removed, and the remaining fruit material was further dried in a ventilated oven (Memmert UF55, Schwabach, Germany; forced air circulation, 100% fan speed) at 40 °C for 4 days to ensure complete dehydration. The dried fruits were then finely ground into powder using a coffee bean mill (Sonifer SF-3508, 160w, Yiwu, China) and passed through a 250 µm mesh sieve to obtain a homogeneous particle size. After drying and grinding, the moisture content of the *Prunus spinosa* fruit powder was determined and found to be 5.39 ± 0.21%, indicating effective drying suitable for subsequent extraction and storage.

### 2.2. Ultrasound-Assisted Extraction (UAE)

Ultrasound-assisted extraction (UAE) was performed following a modified protocol based on Benmanseur et al. [20]. A Sonics Vibra-Cell VCX-130-PB ultrasonic processor (Sonics & Materials Inc., Newtown, CT, USA), equipped with a titanium probe allowing precise control of ultrasonic amplitude and operating cycles. The probe was immersed approximately 2 cm into the extraction medium. For each extraction, 0.25 g of dried *Prunus spinosa* L. fruit powder was mixed with 5 mL of a glycerin–water solvent system, with the glycerin-to-water ratio adjusted according to the experimental design. To limit temperature rise during sonication, the extraction vessel was immersed in an ice–water bath throughout the ultrasound-assisted extraction process, maintaining the temperature below 30 °C. This cooling strategy was applied uniformly to all experiments and to both hydroglyceric and ethanolic solvent systems. Extractions were carried out under controlled conditions by varying water concentration in the glycerin-based solvent, ultrasound amplitude and extraction time, as specified in the optimization study. At the end of sonication, the crude extracts were centrifuged at 4000 rpm for 15 min to remove solid residues. The resulting supernatants were then filtered through Whatman No. 1 filter paper, transferred into sealed vials, and stored at 4 °C and analyzed on the same day. Following the determination of optimal UAE conditions, both hydroglyceric and 70% (*v*/*v*) ethanol extracts were prepared under these conditions, using the same powder-to-solvent ratio (0.5 g/5 mL). For UHPLC-PDA-ESI-HRMS analysis, the extracts were freeze-dried to remove water.

### 2.3. Data Collection for the Box–Behnken Design (BBD)

The selection of independent variables was guided by both equipment-specific constraints and preliminary experimental evaluation. In the probe-type ultrasonic system used in this study, ultrasonic frequency and nominal power are fixed by the manufacturer, whereas ultrasonic amplitude and extraction time represent the only adjustable sonication parameters. Additional variables, such as temperature and solid-to-solvent ratio, were maintained as constant based on preliminary trials and literature evidence indicating negligible effects within typical operational ranges. This strategy allowed the optimization to focus on the most influential factors affecting extraction performance. This approach is consistent with previous UAE optimization studies that proceeded directly to Response Surface Methodology when the number of controllable variables was limited and supported by preliminary experiments and literature evidence [21,22,23,24].

The experimental ranges selected for the Box–Behnken design were defined based on preliminary experiments conducted to identify suitable extraction conditions. Ultrasound time was initially explored between 1 and 12 min, glycerol–water ratios between 0 and 90%, and ultrasonic amplitude between 10% and 100%. Based on these preliminary trials, the experimental matrix was established to capture the full response range, including both the increase and eventual decrease in total phenolic content. The optimization of the ultrasound-assisted extraction process was carried out using a Box–Behnken design (BBD) implemented with JMP software (version 16 Pro, SAS Institute Inc., Cary, NC, USA). The experimental design included three independent variables, each evaluated at three coded levels (−1, 0, and +1, corresponding to low, medium, and high levels, respectively). A total of fifteen experimental runs were generated, including three replicates at the center point to estimate experimental error and assess model adequacy.

The independent variables investigated were water concentration in the glycerin-based solvent system (20%, 40%, and 60%, X_3_), ultrasound amplitude (20%, 60%, and 100%, X_2_), and extraction time (1, 5, and 9 min, X_1_). The response variables selected to evaluate extraction performance were total phenolic content (TPC), total flavonoid content (TFC), and antioxidant activity determined by the DPPH radical scavenging assay (Table 1).

The experimental data were fitted to a second-order polynomial model, expressed as follows (Equation (1)):

(1) $$\gamma =\alpha^{0}+\sum_{i=1}^{3}\alpha_{i}X_{i}+\sum_{i=1}^{3}\alpha_{\mathrm{ii}}{X_{i}}^{2}+\sum_{i=1}^{2}\sum_{j=i+1}^{3}\alpha_{\mathrm{ij}}X_{i}X_{j}$$

where γ represents the predicted response, α_0_ is the intercept term, and α_i_, α_ii_, and α_ij_ correspond to the linear, quadratic, and interaction coefficients, respectively, associated with the independent variables X_i_ and X_j_.

Optimization of extraction conditions was performed using the prediction profiler function in JMP software, aiming to maximize phenolic extraction efficiency. The optimal parameter combination obtained from the model was subsequently validated experimentally by evaluating TPC, TFC, and antioxidant activity (DPPH assay).

### 2.4. Data Collection for ANN Model Setup

The Artificial Neural Network (ANN) model was developed to describe the relationship between the independent extraction variables and the corresponding response variables through a supervised learning process. The ANN architecture was designed to establish non-linear correlations between input and output parameters using experimental data generated from the Box–Behnken design.

The network consisted of three input neurons corresponding to extraction time, ultrasound amplitude, and water concentration in the glycerin-based solvent system, two hidden layers, and three output neurons representing total phenolic content (TPC), total flavonoid content (TFC), and antioxidant activity (DPPH assay). Different transfer functions, including linear, Gaussian, and hyperbolic tangent functions, were evaluated to identify the most suitable configuration for accurate prediction and generalization.

Model training was performed using an iterative learning process with up to 1000 iterations, during which the network weights were adjusted to minimize prediction error. Model performance was assessed by comparing predicted and experimental values using appropriate statistical indicators, allowing evaluation of the predictive capability and generalization performance of the ANN model.

### 2.5. Analytical Determinations

For TPC, TFC, and DPPH assays, extracts obtained during the optimization experiments were analyzed on the same day of extraction to minimize potential degradation of bioactive compounds. For UHPLC-DAD-HRMS/MS analysis, extracts obtained under optimal conditions (40% water in glycerin and 70% ethanol) were freeze-dried to remove water prior to analysis. In the case of the hydroglyceric extract, freeze-drying resulted in water removal only, as glycerol does not yield a solid powder; therefore, the process was applied to concentrate the extract rather than to obtain a dry solid. All spectrophotometric determinations were performed in triplicate, including appropriate reagent blanks and calibration curves.

#### 2.5.1. Total Phenolic Content (TPC)

Total phenolic content (TPC) was determined using the Folin–Ciocalteu spectrophotometric method as reported in our previous study [25]. Assays were conducted using a Varioskan LUX multimode microplate reader (Thermo Fisher Scientific). TPC was estimated from the calibration curve of GA (range 5–200 μg mL^−1^) and the data were expressed as GA equivalents per g of Dry Weight (mg GAE g^−1^ DW, mean ± SD).

#### 2.5.2. Total Flavonoid Content (TFC)

Total flavonoid content (TFC) was determined according to the colorimetric method described by Kim et al. [26]. Briefly, 400 µL of extract, standard solution, or distilled water (blank) was mixed with 120 µL of 5% (*w*/*v*) NaNO_2_ in glass test tubes. After 5 min of reaction, 120 µL of 10% (*w*/*v*) AlCl_3_ solution was added and the mixture was thoroughly vortexed. After an additional 6 min, 800 µL of 1 M NaOH was added to terminate the reaction, and absorbance was immediately measured at 510 nm using a UV–Vis spectrophotometer against the blank. Quantification was performed using a calibration curve prepared with catechin standard solutions in the concentration range of 0–1000 µg/mL. Results were expressed as milligrams of catechin equivalents per gram of dry weight (mg CE/g DW).

#### 2.5.3. DPPH Free Radical Scavenging Activity

Antioxidant activity was evaluated using the 2,2-diphenyl-1-picrylhydrazyl (DPPH) radical scavenging assay, following the method as described by Sharma and Bhat [27] with minor modifications. Briefly, 1450 µL of a 0.06 mM DPPH solution was mixed with 50 µL of *Prunus spinosa* L. extract. Ascorbic acid was used as a positive control, while pure Methanol served as the blank.

The reaction mixtures were incubated for 30 min at room temperature in the dark, after which absorbance was measured at 515 nm using a UV–Vis spectrophotometer. A calibration curve was prepared using ascorbic acid standard solutions.

The DPPH radical scavenging activity was calculated according to Equation (2):

(2) $$Antioxidant\;activity\%=\frac{\left(Acontrol-Asample\right)}{Acontrol}\;\;\times 100$$

where Acontrol represents the absorbance of the negative control and Asamplecorresponds to the absorbance of the tested extract.

#### 2.5.4. UHPLC-DAD-HRMS/MS Characterization of *Prunus spinosa* Extracts

The phytochemical profiles of *Prunus spinosa* extracts were characterized using a Vanquish Flex UHPLC system (Thermo Fisher Scientific, Milan, Italy) coupled to a diode array detector (PDA) and an Orbitrap Exploris 120 high-resolution mass spectrometer equipped with a heated electrospray ionization source (HESI-II). Chromatographic separation was achieved on a Kinetex C18 column (100 × 2.1 mm i.d., 2.6 μm; Phenomenex, Bologna, Italy) using a binary solvent system consisting of water (A) and acetonitrile (B), both acidified with 0.1% (*v*/*v*) formic acid.

The elution gradient was programmed as follows: 0–3 min, 2% B; 3–5 min, 2–8% B; 5–9 min, 8% B; 9–16 min, 8–18% B; 16–17 min, 18% B; 17–21 min, 18–30% B; 21–24 min, 30–98% B; 24–26 min, 98% B. UV–Vis spectra were recorded in the 200–600 nm range.

High-resolution mass spectrometric data were acquired in both positive and negative ionization modes. MS acquisition was performed in Full MS/data-dependent MS^2^ (dd-MS^2^) mode, with resolving powers set at 60,000 and 30,000 FWHM for Full MS and MS^2^ scans, respectively. Fragmentation was induced using stepped higher-energy collisional dissociation (HCD) at collision energies of 20, 40, and 60 eV.

Compound identification was based on the combined evaluation of retention times, UV–Vis spectral characteristics, accurate mass measurements, and MS/MS fragmentation patterns, supported by comparison with literature data. Quality control procedures were applied throughout UHPLC-DAD-HRMS/MS analysis to minimize the risk of false-positive compound annotations. Solvent blanks were analyzed to monitor potential background signals or carryover effects, and only features consistently detected across replicate injections were considered. Compound identification was further supported by the combined agreement of retention behavior, UV–Vis spectra, accurate mass measurements, and MS/MS fragmentation patterns reported in the literature. UHPLC-DAD-HRMS/MS analysis enabled the identification of 29 specialized metabolites in *Prunus spinosa* extracts, mainly belonging to phenolic acids and flavonoid glycosides.

### 2.6. Statistical Analysis

All experiments were performed in triplicate, and results are expressed as mean ± standard deviation (SD). Response Surface Methodology (RSM) and Artificial Neural Network (ANN) models were developed using JMP software (version 16 Pro, SAS Institute Inc., Cary, NC, USA). Statistical significance of differences between glycerin-based extraction and conventional 70% ethanol extraction was evaluated using Student’s *t*-test. Differences were considered statistically significant at *p* < 0.05.

Graphical representation of data was performed using OriginPro 2022 software (OriginLab^®^, Northampton, MA, USA).

## 3. Results

### 3.1. UHPLC-DAD-HRMS/MS Profiling of Prunus spinosa Extracts

**Figure 1:**
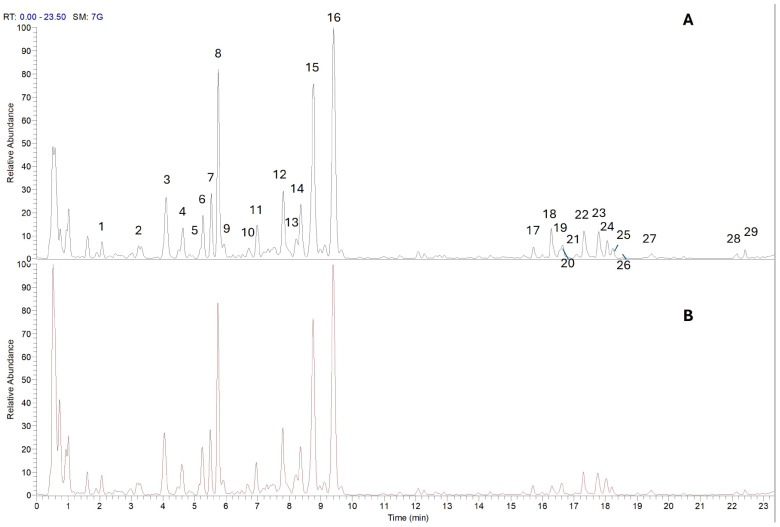
UHPLC-DAD-HRMS/Ms profiles of Ethanolic extract (**A**) and Hydroglyceric extract (**B**).

The phytochemical composition of *Prunus spinosa* fruit extracts obtained using ethanolic and hydroglyceric solvents was investigated by UHPLC-DAD-HRMS/MS analysis. Representative chromatographic profiles of the ethanolic (A) and hydroglyceric (B) extracts are reported in Figure 1, while the identified compounds and their corresponding spectral characteristics are summarized in Table 2.

Overall, UHPLC-HRMS analysis enabled the detection and tentative identification of 29 specialized metabolites in *Prunus spinosa* extracts. Compound identification was achieved by combining accurate mass measurements, MS/MS fragmentation patterns, UV–Vis spectral data, and comparison with literature references, and was assigned according to the Metabolomics Standards Initiative (MSI) guidelines. Most compounds were identified at MSI level 2, with chlorogenic acid unambiguously identified at MSI level 1.

The identified metabolites mainly belonged to phenolic acids and flavonoid derivatives, confirming the polyphenol-rich nature of P. spinosa fruits. Among phenolic acids, several hydroxycinnamic acid derivatives were detected, including chlorogenic acid, caffeoylquinic acid isomers, coumaroylquinic acid, and caffeoylquinic hexosides. Chlorogenic acid was detected, in agreement with reported values of 0.14–0.40 mg/g in *Prunus spinosa* ethanolic extracts [28]. In addition, phenolic acid glycosides such as protocatechuic acid-hexoside and vanillic acid-hexoside were observed, indicating extensive glycosylation within the fruit matrix, which enhances the solubility and stability of these phenolic acids and facilitates their recovery in polar solvents [29].

The flavonoid fraction was dominated by flavonol glycosides, mainly quercetin-based derivatives, including quercetin O-hexose, quercetin O-pentose, quercetin O-hexose-deoxyhexose, and quercetin O-pentose-hexose. Isorhamnetin and luteolin glycosides were also detected, highlighting the structural diversity of flavonoids present in the extracts. Additionally, minor constituents such as benzyl β-primeveroside, ionone-hexoside, and acylated sugar derivatives were identified, together with a limited number of unknown compounds tentatively classified based on their mass spectral features.

From a qualitative perspective, the UHPLC-DAD-HRMS/MS profiles of ethanolic and hydroglyceric extracts were largely overlapping, showing a comparable distribution of the detected compounds. The identified metabolites mainly included hydroxycinnamic acid derivatives, caffeoylquinic acid isomers, and flavonol derivatives. UHPLC-DAD-HRMS/MS analysis was therefore employed as a qualitative tool to characterize the overall phytochemical profile of the extracts. Given the complex phytochemical nature of the matrices and the lack of available reference standards, targeted quantitative analysis at the individual compound level was not pursued. Quantitative comparison of extraction efficiency between glycerin and ethanol was instead assessed using spectrophotometric assays, namely TPC and TFC, which provide a semi-quantitative evaluation of major classes of bioactive compounds in plant extracts. The similarity of the qualitative profiles indicates that ultrasound-assisted extraction enabled the recovery of comparable classes of bioactive compounds from *Prunus spinosa* fruits using both solvent systems. Importantly, despite the qualitative similarity between the two profiles, the ability to obtain comparable phytochemical fingerprints using glycerine, a food-grade, environmentally friendly solvent, represents a relevant and encouraging outcome. This finding indicates that glycerin-based ultrasound-assisted extraction can effectively replace conventional ethanolic extraction, which is commonly employed for the production of standardized extracts and dietary supplements, without compromising the qualitative composition of the recovered bioactive compounds. Although no marked qualitative differences were observed between ethanolic and hydroglyceric extracts, the ability to obtain an essentially overlapping phytochemical fingerprint using a food-grade, environmentally benign solvent represents a relevant advantage for the development of safe and sustainable extracts intended for food and nutraceutical applications. The lack of compound-level quantitative data represents a limitation of the present study; however, the combined use of spectrophotometric assays and qualitative UHPLC–HRMS profiling provides a robust comparative assessment of extraction efficiency at the phytochemical class level. While a deeper characterization of physicochemical and functional properties (e.g., solubility, stability, rheological behavior, or techno-functional performance) would further support application-oriented development, such analyses were beyond the scope of the present work, which was specifically designed to optimize extraction conditions and evaluate modeling performance. These aspects deserve dedicated investigation in future studies. In addition, physicochemical and structural characterizations (e.g., particle size/zeta potential, FT-IR or SEM) were not considered, as the study did not aim to characterize colloidal or material properties of the extracts, but rather their phytochemical composition and extraction behavior.

### 3.2. Performance of RSM and ANN Model

In this study, a Box–Behnken Design (BBD) was used as the experimental design to generate the extraction runs. Response Surface Methodology (RSM) was used to fit quadratic polynomial equations, whereas an Artificial Neural Network (ANN) was employed as a complementary predictive approach to investigate the relationships between the extraction variables, sonication time (X_1_), ultrasound amplitude (X_2_), and water concentration in glycerin (X_3_), and the measured responses, namely total phenolic content (TPC), total flavonoid content (TFC), and antioxidant activity (DPPH assay). The experimental and predicted values obtained from both modeling approaches are reported in Table 3.

Overall, a strong agreement between experimental and predicted values was observed for all responses, indicating that both RSM and ANN were suitable for describing the extraction process. The close correspondence between observed and predicted responses confirms the adequacy of the regression models for optimization purposes, in agreement with previous studies applying hybrid statistical–intelligent approaches to extraction processes [12]. Model validity was initially assessed through determination coefficients (R^2^), which showed high values close to unity for all responses, reflecting a strong correlation between experimental data and model predictions. Additional statistical parameters, including RMSE, F-ratio, *p*-values, mean square, and sum of squares, were used to further evaluate model quality (Table 4). Analysis of variance (ANOVA) confirmed the statistical significance of the developed models and highlighted the contribution of linear, quadratic, and interaction terms (Table 5). The adequacy of the RSM models was further confirmed by non-significant lack-of-fit values (*p* > 0.05), indicating that the quadratic models were sufficient to describe the experimental data within the investigated domain. High adjusted R^2^ values, together with low RMSE and SSE, confirm that the models were not overfitted and retained good predictive capability. The response surface plots revealed clear curvature effects and interaction patterns among extraction time, ultrasonic amplitude, and water concentration in glycerin. Similar interpretations of surface curvature, interaction strength, and slope steepness in ultrasound-assisted extraction processes have been reported for polyphenol-rich plant matrices and biomass systems, where cavitation-controlled mass transfer and solvent physicochemical properties govern non-linear extraction behavior [23,24,30]. In particular, the dome-shaped surfaces observed for TPC and TFC indicate the presence of an optimal operational region rather than a monotonic increase. This behavior reflects the balance between enhanced mass transfer induced by acoustic cavitation at moderate water contents and potential dilution or matrix effects at higher water levels. These trends are in line with the physicochemical behavior of glycerol–water systems under ultrasonic fields and support the mechanistic interpretation of the observed extraction responses. Overall, the observed non-linear trends can be rationalized by the combined effects of reduced solvent viscosity and improved cavitation-driven mass transfer. Notably, the presence of an optimal region at intermediate water content reflects a balance between enhanced cavitation efficiency and dilution or matrix effects at higher water levels. Similar non-linear behaviors and optimal intermediate solvent compositions have been reported for ultrasound-assisted extraction of polyphenols from other plant matrices, supporting the mechanistic interpretation proposed in the present study. While ANN showed comparable predictive accuracy, RSM was retained as the primary optimization tool due to its higher interpretability and explicit representation of variable interactions.

#### 3.2.1. The RSM and ANN Modeling for (TPC)

**Figure 2:**
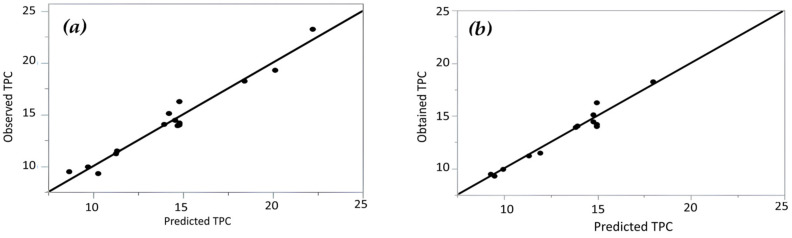
Observed versus predicted total phenolic content (TPC) values obtained using (**a**) Response Surface Methodology (RSM) and (**b**) Artificial Neural Network (ANN) models.

According to the ANOVA results (Table 3), the RSM model developed for TPC showed an excellent fit, with an R^2^ value of 0.96, indicating that more than 96% of the variability in TPC could be explained by the selected extraction variables [31]. The lack-of-fit test was not statistically significant (*p* > 0.05), confirming that the model adequately described the experimental data. The strong agreement between predicted and experimental TPC values is also illustrated in Figure 2. Although a high R^2^ value generally indicates good model performance, it may be artificially inflated by the inclusion of non-significant terms. Therefore, the adjusted R^2^ (adj-R^2^) was also considered to evaluate model robustness [32]. The small difference observed between R^2^ and adj-R^2^ values suggests that non-significant variables did not meaningfully contribute to the model, confirming its statistical reliability. Recent studies have reported comparable results, where RSM and ANN approaches were applied to optimize microwave-assisted extraction of phenolics from mango peel, achieving high predictive accuracy and reliable models [33]. Comparative analyses also showed that ANN models frequently capture nonlinear interactions among variables more accurately than RSM, particularly in complex extraction systems of bioactive compounds [34]. Additionally, the combined use of RSM and ANN has been identified as an effective strategy for both process optimization and bioactivity evaluation in phenolic-rich matrices such as *Pithecellobium dulce* fruit peels [35]. The second-order polynomial equation describing the effect of extraction variables on TPC is presented in Equation (3):

(3) $${\;\gamma }_{TPC}=13.74+2.43X_{1}+3.73X_{2}+1.82\;X_{1}X_{2}-1.71X_{2}X_{3}+2.2\;X_{2}^{2}$$

Based on the model coefficients (Table 4), TPC was mainly influenced by extraction time, ultrasound amplitude, the interaction between these two factors, and the quadratic effect of ultrasound amplitude. These findings are in agreement with previous reports showing that increasing ultrasonic amplitude significantly enhances polyphenol extraction efficiency [36]. Similar interpretations of surface curvature, interaction strength, and slope steepness in ultrasound-assisted extraction processes have been reported for polyphenol-rich matrices and biomass systems, where cavitation-controlled mass transfer and solvent physicochemical properties govern non-linear extraction behavior [23,24,30]. Furthermore, as noted by [37], the use of glycerin-water mixtures as a green solvent significantly affects extraction efficiency. While pure glycerin is highly viscous, the incorporation of ~20% water markedly lowers viscosity and surface tension, promoting more efficient acoustic cavitation and improving energy transfer during ultrasound-assisted extraction. Under these conditions, ultrasound propagation generates intense pressure gradients within the medium, leading to the formation of cavitation microbubbles. These bubbles collapse violently, generating high-shear forces and micro-jets that cause several effects: (i) cell wall disruption through mechanical breakdown of the plant matrix, releasing bound phenolics; (ii) enhanced permeability by increasing tissue swelling, allowing deeper solvent penetration; and (iii) accelerated mass transfer through rapid diffusion of intracellular compounds into the glycerin-water mixture. This increased kinetic energy overcomes the mass transfer resistance typically associated with viscous polyol-based solvents [38]. The optimal extraction conditions predicted by the RSM model were 20% water content in glycerin, 100% ultrasound amplitude, and 9 min extraction time, under which a maximum TPC of 24.09 mg GAE/g DW was predicted. This value is in close agreement with experimentally validated results, confirming the robustness of the optimization strategy.

#### 3.2.2. The RSM and ANN Models for TFC

For Response Surface Methodology (RSM) model applied to total flavonoid content (TFC), statistical analysis revealed significant linear effects of extraction time (X_1_) and ultrasonic amplitude (X_2_), along with a significant interaction between these two factors (X_1_X_2_) and significant quadratic terms (X_1_^2^ and X_3_^2^). Based on the polynomial regression model (Equation (4)), the optimal extraction conditions were identified as 9 min extraction time, 100% ultrasonic amplitude, and 37.27% water content in glycerin. Under these conditions, the predicted TFC value reached 20.36 mg CE/g DW.

The model exhibited a high coefficient of determination (R^2^ = 0.95), indicating that more than 95% of the variability in TFC was explained by the selected independent variables, while only 5% of the total variation remained unexplained [31].

**Figure 3:**
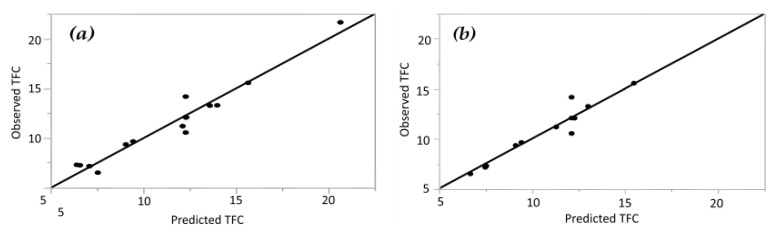
Observed versus predicted total flavonoid content (TFC) values obtained using (**a**) Response Surface Methodology (RSM) and (**b**) Artificial Neural Network (ANN) models.

This high level of reliability is in line with the findings of Akpabli-Tsigbe et al. [37], who reported comparable model robustness for phenolic-related responses during solid-state fermentation–based optimization studies. Moreover, the lack-of-fit test was not significant at the 95% confidence level, confirming that the proposed model adequately represented the experimental data. As observed for the TPC model, the adjusted R^2^ value was also considered to ensure model robustness, confirming that non-significant variables were excluded from the final equation [32]. A strong agreement between predicted and experimental values further supported model reliability (Figure 3).

(4) $${\;\gamma }_{TFC}=14.11+2.21X_{1}+3.81X_{2}+1.66\;X_{1}X_{2}-1.67X_{1}^{2}-2.43\;X_{3}^{2}$$

These results indicate that TFC extraction is primarily governed by extraction time, ultrasonic amplitude, and their interaction, with additional contributions from quadratic effects related to amplitude and water content. High ultrasonic amplitude (100%) significantly enhanced flavonoid recovery, in agreement with previous findings reporting increased cavitation intensity and improved cell wall disruption at higher amplitudes [39].

Regarding solvent composition, an intermediate water concentration (~35–40%) in glycerin was found to be optimal for flavonoid extraction. This water content reduces solvent viscosity, improving mass transfer and penetration into the plant matrix, while preserving sufficient solvent polarity to solubilize flavonoids. Excessive water addition may reduce flavonoid solubility, whereas insufficient water limits diffusion and accelerates solvent saturation.

For the ANN model, predicted and experimental TFC values showed excellent agreement (Table 3). The ANN demonstrated high predictive accuracy, with R^2^ values of 0.93 and 0.98 for the training and validation datasets, respectively (Table 5). Error analysis further confirmed ANN robustness, with RMSE values of 0.73 (training) and 0.52 (validation), which were lower than those obtained using the RSM model (RMSE = 1.51).

Overall, both models provided reliable predictions for TFC extraction; however, ANN exhibited slightly higher flexibility in capturing non-linear relationships within the extraction system.

#### 3.2.3. The RSM and ANN Models for DPPH Radical Scavenging Activity

The antioxidant activity, expressed as DPPH radical scavenging activity (RSA), was strongly influenced by the extraction parameters, making it a critical response for process optimization. For the RSM model, significant effects (*p* < 0.05) were observed for ultrasonic amplitude (X_2_), interaction terms (X_1_X_2_ and X_1_X_3_), and quadratic terms (X_1_^2^ and X_2_^2^).

Using the polynomial regression model (Equation (5)), the optimal extraction conditions for maximizing RSA were identified as 9 min extraction time, 100% ultrasonic amplitude, and 60% water content in glycerin. Under these conditions, the predicted RSA reached 92.63%, indicating strong free radical scavenging capacity. This enhancement is largely attributed to the cavitation- and mechanically assisted release of free-form phenolic acids from weakly bound macromolecular complexes (e.g., proteins and polysaccharides) within the plant cell wall [37].

(5) $${\;\gamma }_{DPPH}=58.76+4.34X_{1}+4.21{X_{1}X}_{2}+4.12\;X_{1}X_{3}+5.08X_{1}^{2}+14.17\;X_{2}^{2}$$

The (RSM) model exhibited a high R^2^ value of 0.96, confirming that the selected variables explained more than 96% of the variability in RSA. The adjusted R^2^ further supported model reliability, indicating that non-significant terms were effectively excluded. These results are in agreement with recent bioprocess optimization studies where R^2^ values exceeding 0.73 were deemed indicative of adequate and significant regression models for tracking bioactive responses [37]. A strong correlation between predicted and experimental values was also confirmed (Figure 3).

For the ANN model, RSA prediction accuracy was high, with R^2^ values of 0.98 and 0.85 for the training and validation datasets, respectively (Table 5). Although a slight reduction in validation performance was observed, attributed to the limited number of experimental runs, the ANN predictions remained consistent with RSM outputs. Error metrics (RMSE, MSE, MAE, and MAPE) confirmed that both models were reliable, with ANN showing improved performance in describing the non-linear behavior of antioxidant activity. The flexibility of the ANN model in this study reflects the complexity of biochemical interactions, where synergistic metabolic effects and structural modifications often result in non-linear relationships that traditional first or second-order equations may not fully capture [33].

### 3.3. ANN Modeling and Comparison with RSM

The Artificial Neural Network (ANN) model was applied to predict total phenolic content (TPC), total flavonoid content (TFC), and antioxidant activity, and its performance was directly compared with that of the RSM model (Table 6). For all responses, a high degree of agreement was observed between experimental and ANN-predicted values, indicating effective learning and strong generalization capability of the ANN model.

For TPC prediction, the ANN achieved R^2^ values of 0.95 and 0.99 for training and validation datasets, respectively, which were comparable to or slightly higher than those obtained using the RSM model. Similar trends were observed for TFC and antioxidant activity. Error analysis indicated slightly lower RMSE and MSE values for ANN, while both models exhibited robust predictive capabilities within the experimental design. Specifically, RMSE values decreased from 1.27 (RSM) to 0.56 and 0.12 for ANN training and validation, respectively.

These results highlight the ability of ANN to effectively capture complex and non-linear relationships among extraction variables, even when based on a limited experimental dataset. According to Ejimofor et al. [40] RMSE values below 3 indicate reliable predictive performance, further confirming the robustness of the developed ANN models. Although metrics such as MAE provide complementary information, the low MAPE values obtained for both modeling approaches further support the overall predictive accuracy, with ANN showing a slight advantage over RSM in describing the extraction process.

### 3.4. Analysis of Response Surface Plots for ANN and RSM Models

**Figure 4:**
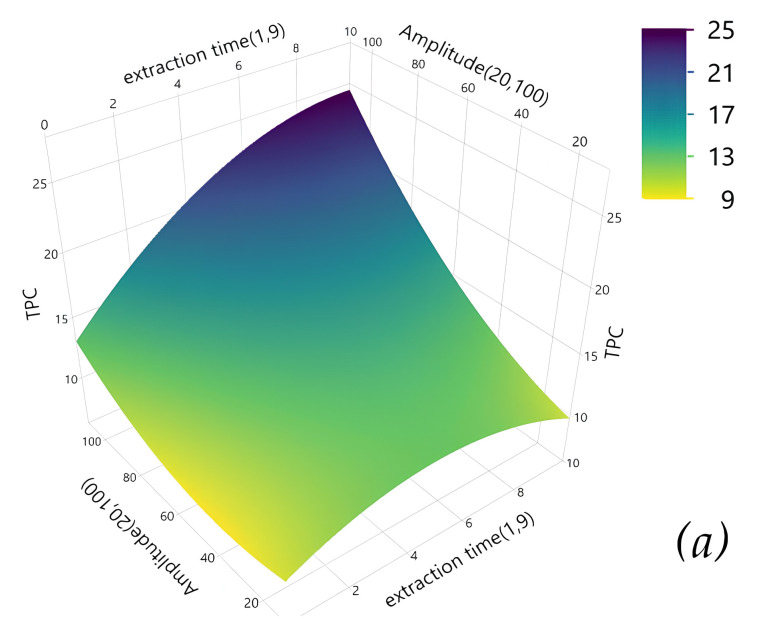

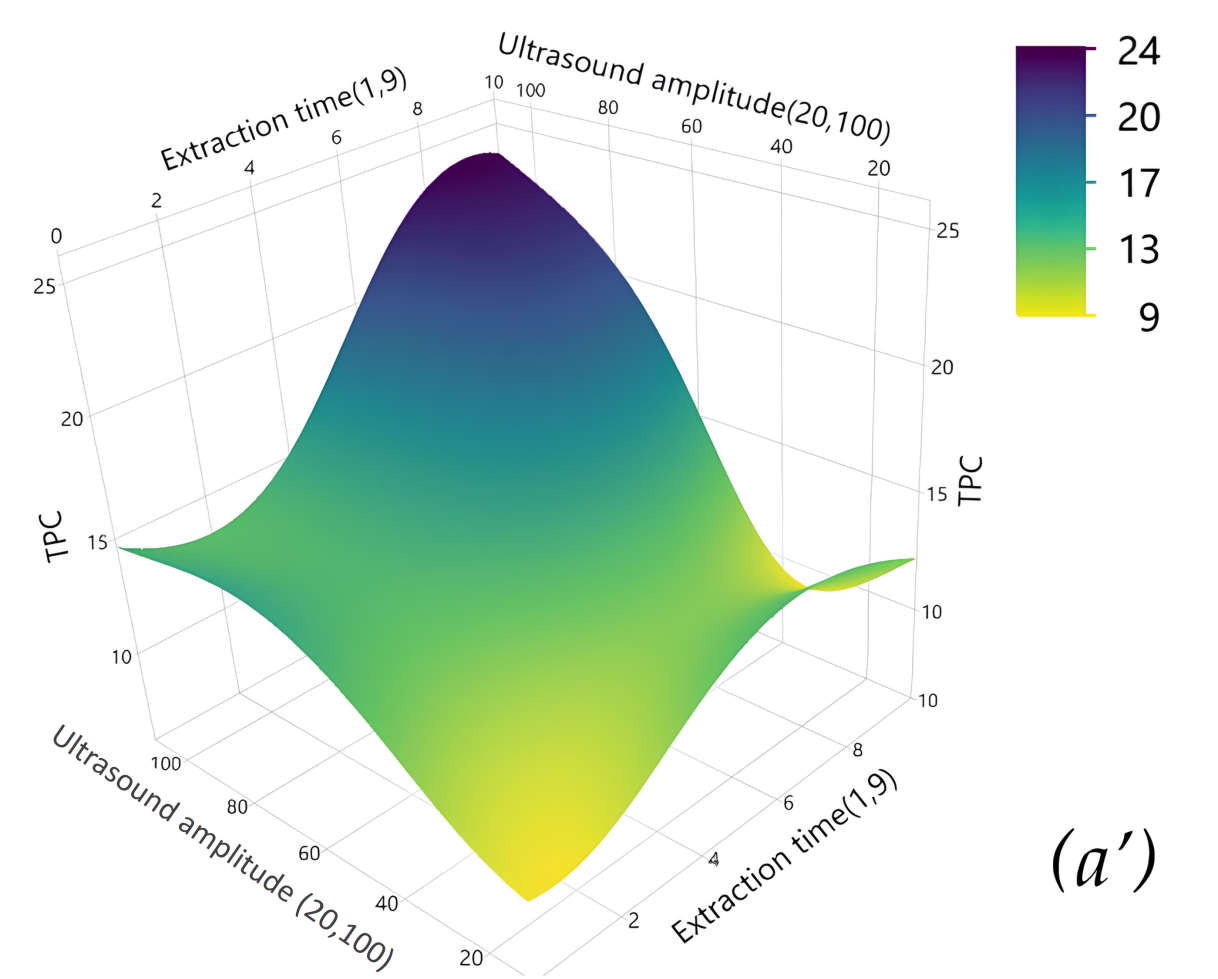

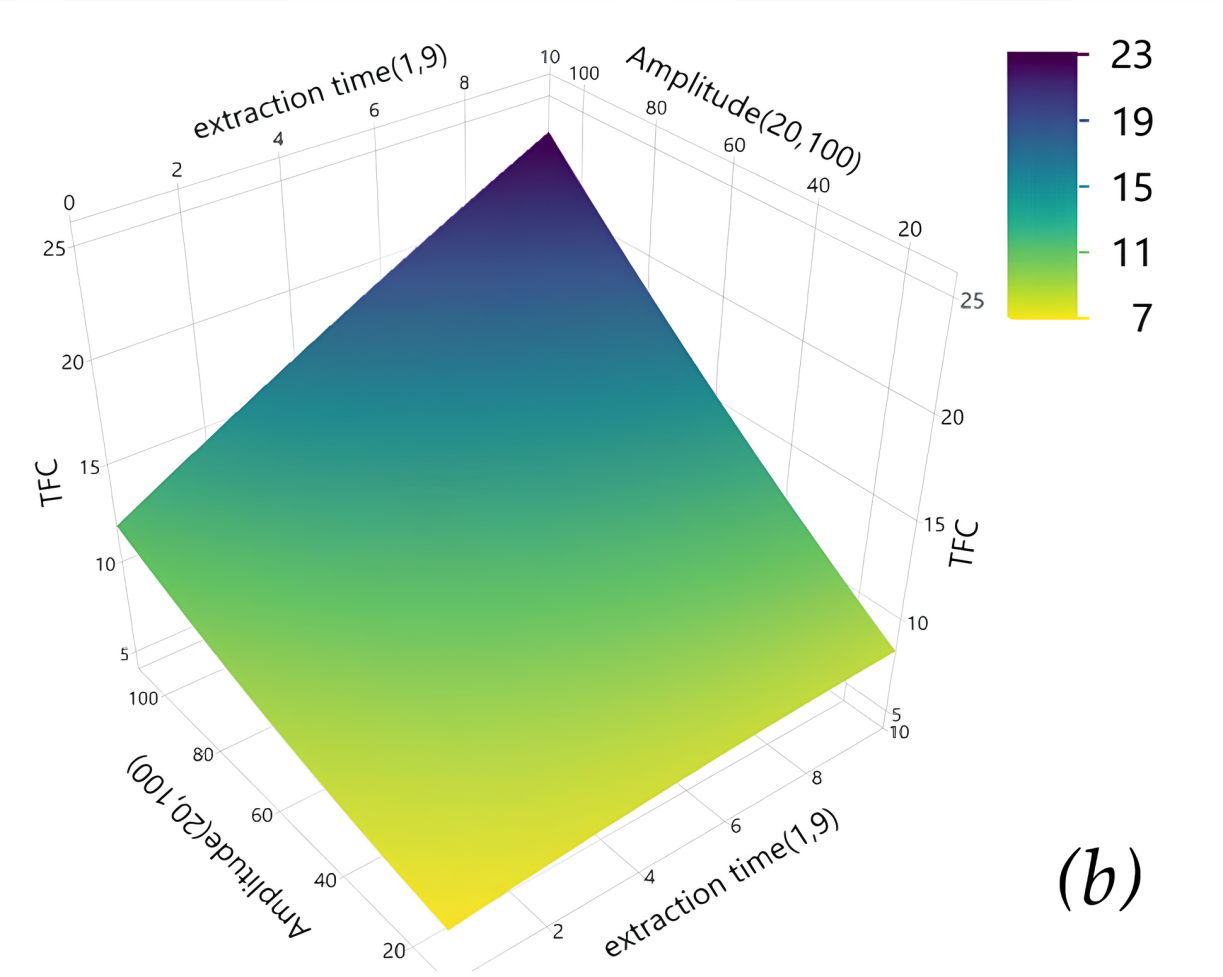

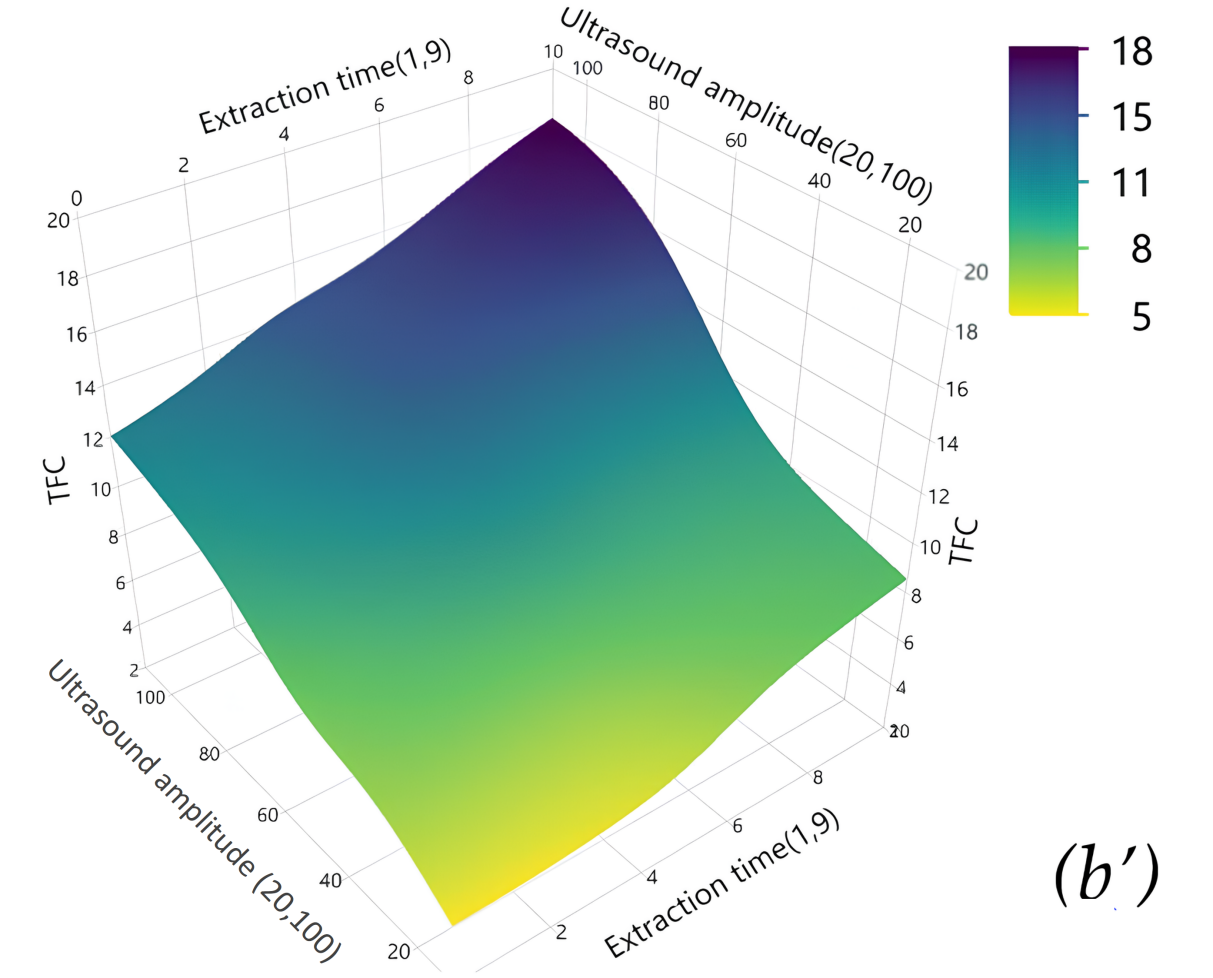

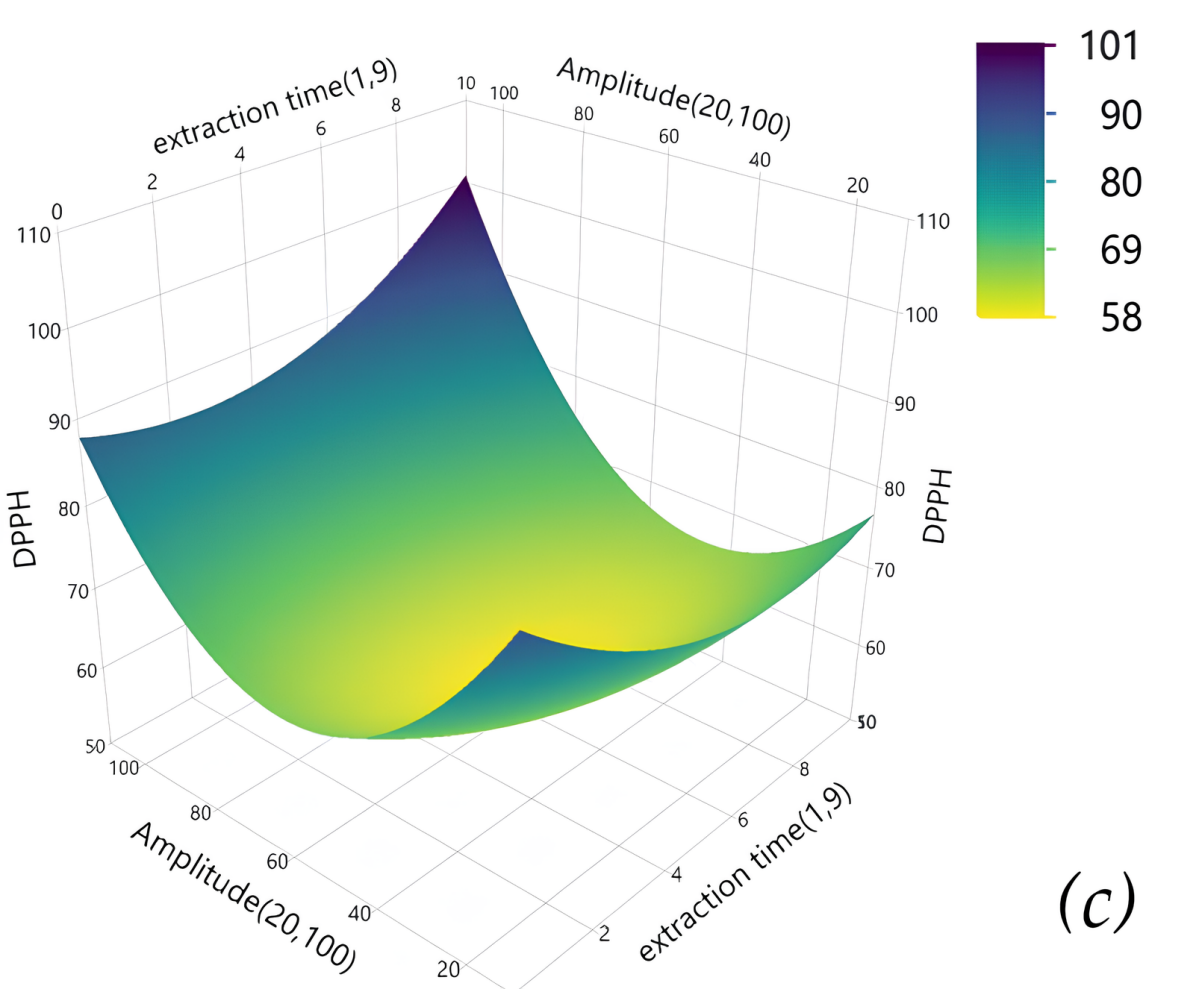

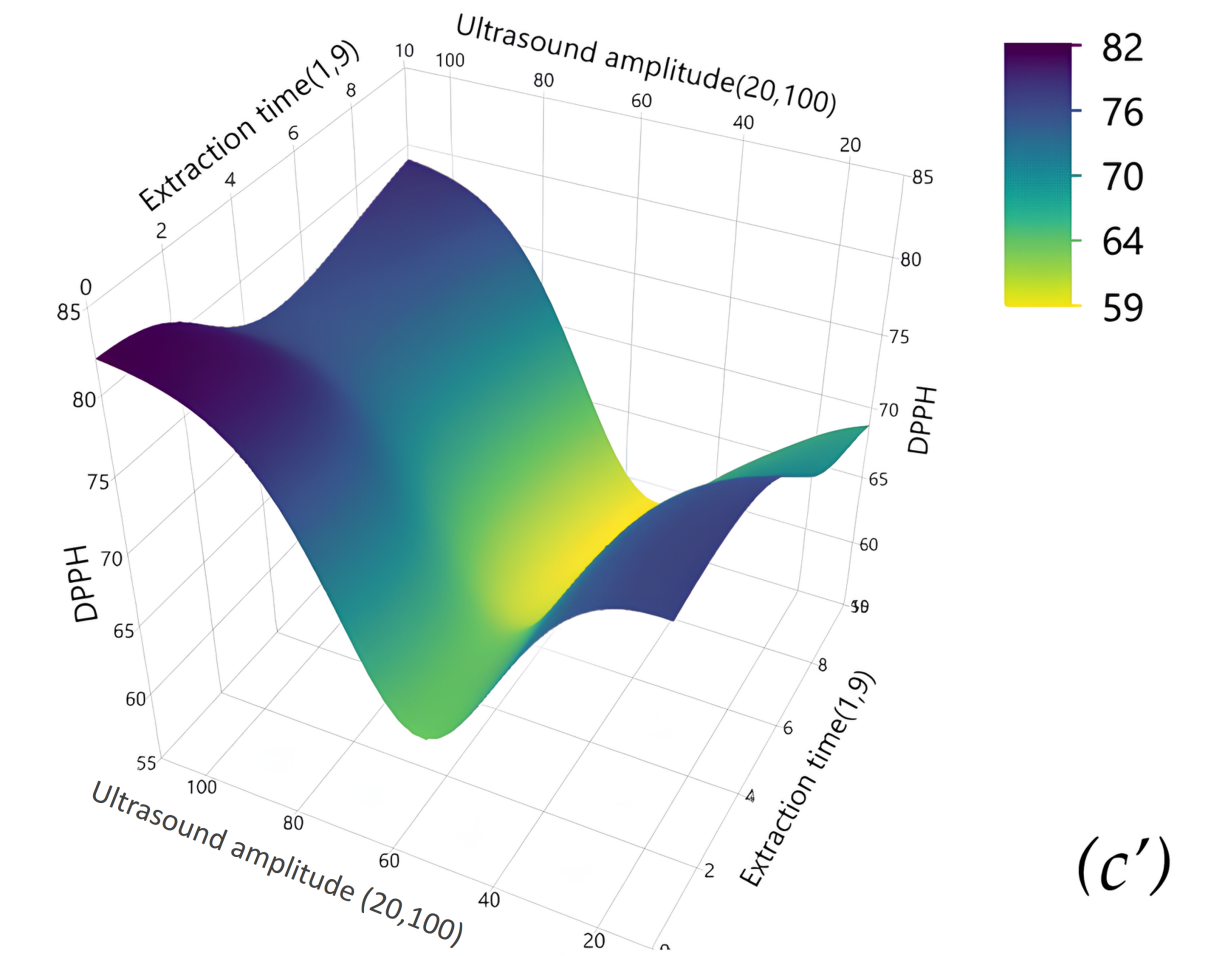
Interactive effects of extraction variables on (**a**,**a′**) total phenolic content (TPC), (**b**,**b′**) total flavonoid content (TFC), and (**c**,**c′**) antioxidant activity (RSA) as predicted by the RSM and ANN models, respectively.

Three-dimensional response surface plots were generated to visualize the interactive effects of extraction variables, ultrasonic time, ultrasound amplitude, and water concentration, on total phenolic content (TPC), total flavonoid content (TFC), and antioxidant activity (RSA). These plots illustrate the non-linear relationships between independent variables and responses for both Box–Behnken Design (RSM) and Artificial Neural Network (ANN) models (Figure 4). The response surface analysis demonstrates the complementary nature of RSM and ANN approaches in capturing interactive effects between extraction parameters.

Taken together, the interaction trends obtained using the RSM model were comparable to those predicted by the ANN model. However, the ANN response surface plots exhibited additional curvature for TPC (Figure 4a′), TFC (Figure 4b′), and RSA (Figure 4c′), reflecting the ability of ANN to capture complex non-linear behavior and to extrapolate beyond the experimental design space in identifying global optima.

For TPC, both models show smooth, gradual surfaces with moderate curvature (Figure 4a,a’), indicating relatively predictable relationships between variables, and showed a gradual increase with increasing extraction time and ultrasound amplitude, reaching maximum values at 9 min of sonication. However, the surfaces for TFC (Figure 4b,b’) display more pronounced curvatures with noticeable gradients, particularly in the ANN model which captures additional non-linear patterns. Extraction yield increased with time and amplitude, with optimal recovery also occurring at 9 min. This extraction time appears advantageous for preserving flavonoid integrity, as prolonged ultrasonic exposure may lead to partial degradation of antioxidant compounds [41]. The response surface plots further indicate that TPC and TFC followed comparable extraction patterns, mainly driven by ultrasound amplitude and extraction time. These findings are consistent with the ANOVA results, which identified ultrasound amplitude as a dominant factor influencing extraction efficiency. Increasing amplitude intensifies acoustic cavitation by increasing the number of compression and rarefaction cycles, thereby enhancing cell wall disruption and mass transfer of solutes into the solvent [42]. As a result, polyphenols and flavonoids are rapidly released during the initial extraction phase [43]. After this stage, extraction rates tend to decrease as the remaining compounds are more tightly bound within the plant matrix. Regarding ultrasonic time, TPC and TFC reached their maximum values within a relatively short extraction duration (9 min). This behavior suggests that *Prunus spinosa* L. releases its phenolic and flavonoid compounds rapidly, leading to early saturation of the extraction medium. Prolonged sonication may promote degradation or emulsification of flavonoids, resulting in lower net recovery [44]. Therefore, short-term sonication appears more suitable for efficient flavonoid extraction from *P. spinosa* fruits. In terms of antioxidant activity, phenolic content and radical scavenging capacity are generally reported to be positively correlated [45]. Nevertheless, in the present study, RSA (Figure 4c,c’) exhibits more complex surface topology with visible peaks and depressions, especially in the ANN predictions, suggesting non-linear interactions where the combined effect of extraction variables influences antioxidant activity in ways that the ANN model captures with more detail than the polynomial RSM approach. As shown in Figure 4c′, antioxidant activity initially decreased and subsequently increased with increasing ultrasound amplitude and extraction time. This behavior was more clearly captured by the ANN model, highlighting its suitability for modeling non-linear dynamics. Moderate ultrasound amplitudes promote cavitation and enhance the release of antioxidant compounds, whereas excessive ultrasonic energy may induce degradation of sensitive molecules such as flavonoids or vitamin C. At higher energy input, however, the formation of new antioxidant-active compounds has been reported, potentially explaining the observed recovery in antioxidant activity [46].

### 3.5. Comparison of RSM and ANN Models

The predictive performance of RSM and ANN models was evaluated using multiple statistical indicators, including R^2^, MAE, MAPE, RMSE, and MSE. For all responses, both modeling approaches demonstrated high accuracy, with R^2^ values of 0.96 and 0.95 for TPC, 0.95 and 0.93 for TFC, and 0.96 and 0.98 for RSA for RSM and ANN models, respectively.

Although both approaches provided reliable predictions, the ANN model exhibited slightly better performance in capturing non-linear relationships, particularly evident in the graphical representation of response surfaces. However, given the limited size of the experimental dataset derived from the Box–Behnken design (15 runs), ANN predictions should be interpreted within the investigated design space and should not be intended for broad generalization beyond the experimental domain. Low MAE and MAPE values for both models indicate minimal deviation between predicted and experimental values, confirming strong predictive reliability. Moreover, the slightly lower RMSE values obtained for ANN reflect comparable predictive performance between the two models.

In this context, ANN should be interpreted as a complementary modeling tool rather than a replacement for RSM, providing additional insight into potential non-linear behaviors within the explored experimental domain. Taken together, these results demonstrate that while RSM remains a robust and interpretable optimization tool, ANN offers increased flexibility for modeling complex extraction systems characterized by non-linear interactions.

### 3.6. Validation and Generalization of Optimal Extraction Conditions

The optimal extraction conditions were determined by maximizing the overall desirability function using the JMP prediction profiler, resulting in an extraction time of 9 min, 100% ultrasound amplitude, and 40% water content in glycerin. The use of a desirability-based multi-response optimization approach is well established in RSM studies dealing with the simultaneous maximization of extraction yield and antioxidant activity [22,23,24], and allows the concurrent optimization of TPC, TFC, and antioxidant activity. Under these optimal conditions, the predicted values were TPC = 22.22 mg GAE/g DW, TFC = 20.64 mg CE/g DW, and RSA = 86.57% for the RSM model, and TPC = 22.92 mg GAE/g DW, TFC = 21.61 mg CE/g DW, and RSA = 86.14% for the ANN model, with composite desirability indices of 0.86 and 0.66, respectively. Both models demonstrated acceptable optimization performance, with the RSM model showing higher desirability and the ANN model achieving satisfactory results, confirming that the predicted optimal conditions are reliable for experimental validation.

Experimental validation of these conditions yielded TPC = 21.16 mg GAE/g DW, TFC = 19.77 mg CE/g DW, and RSA = 81.03%, showing good agreement with the predicted values and confirming the reliability of the optimization models. The relative standard error (RSE) between experimental and predicted values was also calculated, with values below 10% for all responses (RSM: 5.01% TPC, 4.45% TFC, 6.84% RSA; ANN: 8.31% TPC, 9.31% TFC, 6.31% RSA), further demonstrating the accuracy and predictive capability of both RSM and ANN models.

The polyphenol yield obtained in this study is comparable to that reported by Veličković et al. [47], who achieved 19.98 ± 1.28 mg GAE/g DW using a 24 h maceration in 96% ethanol combined with ultrasonication (1 h at the beginning and end of the process). Despite the similarity in yield, the present approach offers distinct advantages, as it relies on a natural, food-grade solvent and a significantly shorter extraction time, which could contribute to a safer and more environmentally friendly process. These characteristics align well with the principles of green chemistry, including the potential for reduced energy consumption, minimization of unit operations, and avoidance of thermal treatment [48]. Although studies on *Prunus spinosa* fruits remain limited, some comparative data on flavonoid content are available. Nistor et al. [49] reported a flavonoid content of 20.30 ± 0.17 mg CE/g DW in fruit pulp extracted using 70% ethanol and 30 min of ultrasonication at 40 °C. In comparison, the present study achieved a comparable TFC value (19.77 mg CE/g DW) using the whole fruit (pulp and cuticle), a glycerin-based solvent, a shorter extraction time, and without thermal input. These differences should be interpreted with caution, as fruit origin, harvesting conditions, and environmental factors may influence phytochemical composition. Regarding phenolic content, previous ultrasound-assisted extraction studies provide further context. Drăghici-Popa et al. [50] reported an optimal ultrasound amplitude of 50% with an extraction time of 10 min, close to the conditions found in our study, achieving a TPC of 14.45 ± 0.718 mg GAE/g DM. Similarly Damar & Yilmaz [51], found an optimal extraction time of 3.6 min with an amplitude of 33%. The differences with our results can be attributed to the solvent used; in our study, a hydroglyceric solvent was applied, which has distinct physicochemical properties compared to conventional organic solvents, notably a higher viscosity, affecting the extraction kinetics and efficiency of polyphenols.

### 3.7. Validation of Glycerin Effectiveness for Bioactive Compound Extraction

**Figure 5:**
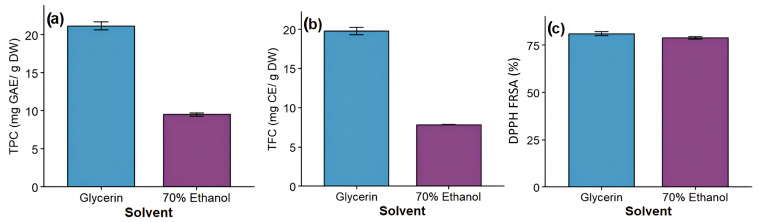
Extraction yields of (**a**) total phenolic content (TPC), (**b**) total flavonoid content (TFC), and (**c**) DPPH radical scavenging activity (%) from *Prunus spinosa* L. fruits obtained using glycerin and 70% ethanol as extraction solvents.

To validate the effectiveness of glycerin as a green extraction solvent, a comparative extraction was performed using conventional 70% ethanol under identical optimized conditions. Differences between solvent systems were statistically evaluated using Student’s *t*-test, and the results are summarized in Table 7. Graphical comparison of extraction performance is presented in Figure 5.

Statistical analysis revealed significantly higher (*p* < 0.05) total phenolic content (TPC), total flavonoid content (TFC), and DPPH radical scavenging activity for glycerin-based extracts compared to those obtained using 70% ethanol. The high *t*-ratio values observed for TPC and TFC further confirm the statistically robust difference between the two solvent systems, indicating a more effective recovery of bioactive compounds when glycerin is employed.

Specifically, glycerin extraction yielded 21.16 ± 0.52 mg GAE/g DW of phenolics and 19.77 ± 0.45 mg CE/g DW of flavonoids, whereas ethanol extraction resulted in significantly lower values (9.49 ± 0.21 mg GAE/g DW and 7.84 ± 0.02 mg CE/g DW, respectively). Antioxidant activity also showed a statistically significant improvement, with glycerin extracts reaching 81.03 ± 1.07% inhibition compared to 78.86 ± 0.82% for ethanol extracts.

Although the use of glycerin as an extraction solvent for bioactive compounds remains relatively underexplored, several studies have demonstrated its effectiveness across different plant matrices, including thanaka [52], lotus seed pod [53], heartwing sorrel [54] and red grape pomace [55]. These findings are in line with the present results and support the suitability of glycerin for polyphenol extraction from *Prunus spinosa* fruits. Beyond extraction performance, glycerin offers additional advantages as a sustainable alternative to conventional organic solvents, including low toxicity, cost-effectiveness, stabilizing properties for bioactive compounds, and direct applicability in food, nutraceutical, cosmetic, and pharmaceutical formulations.

The findings from these studies align with and strongly support our results, consistently demonstrating the effectiveness of glycerin for extraction across different plant matrices, thereby reinforcing the validity of our observations on sloe berries.

Beyond its extraction efficiency, glycerin presents multiple advantages as an eco-friendly alternative to conventional toxic solvents. Its beneficial properties include cost-effectiveness, excellent stabilizing capabilities for bioactive compounds, and safety for incorporation into food, cosmetic, pharmaceutical, and food additive formulations [56].

## 4. Conclusions

This study successfully optimized the extraction of bioactive compounds from *Prunus spinosa* L. fruits by integrating ultrasound-assisted extraction with both Response Surface Methodology (RSM) and Artificial Neural Network (ANN) modeling approaches. The combined use of these tools enabled an accurate evaluation of the effects of key process variables, particularly ultrasound amplitude and extraction time, on the recovery of polyphenols, flavonoids, and antioxidant activity. Both RSM and ANN performed well for process optimization. RSM provided excellent performance with clear interpretability through explicit polynomial equations, while ANN exhibited marginally lower error metrics and additional flexibility in capturing non-linear extraction dynamics. Within the 15 Box–Behnken experimental design, both models demonstrated robust and comparable predictive performance, confirming their complementary suitability for guiding extraction process optimization. Under the optimized conditions, glycerin-based extraction yielded extracts rich in phenolic and flavonoid compounds and exhibiting strong antioxidant activity, as further supported by UHPLC-HRMS phytochemical profiling. The use of glycerin as a food-grade, eco-friendly solvent represents a sustainable alternative to conventional organic solvents, aligning with green chemistry principles while enabling direct applicability of the extracts. Overall, these findings highlight *Prunus spinosa* L. as a valuable and underexploited source of bioactive compounds and demonstrate the effectiveness of combining statistical and artificial intelligence tools for the development of efficient, sustainable extraction processes. Future research should investigate scale-up of the extraction process and evaluate the biological activity of the extracts in in vivo studies. The proposed approach offers promising perspectives for the production of functional ingredients applicable in food, nutraceutical, cosmetic, and pharmaceutical sectors.

## Figures and Tables

**Table 1:** Box–Behnken design variables and their corresponding coded and actual levels.

Factor	Variable	−1 (Low)	0 (Center)	+1 (High)
X_1_	Extraction time (min)	1	5	9
X_2_	Ultrasound amplitude (%)	20	60	100
X_3_	Water concentration (%)	20	40	60

**Table 2:** UHPLC-DAD-HRMS/MS data of compounds detected in Ethanolic extract and Hydroglyceric extract.

N°	Rt	Compound	Formula	Adduct m/z	[M-H]^−^	Product Ion (m/z)	MSI Level ^a^
1	2.07	Isotachoside	C_13_H_18_O_8_	347.0980	301.0932	138, 123	2
2	3.24	Protocatechuic acid-hexoside	C_13_H_16_O_9_		315.0725	153, 109	2
3	4.09	unknown	C_16_H_19_O_9_N		368.0984		
4	4.63	Protocatechuic acid-hexoside	C_13_H_16_O_9_	361.11362	315.0725	153, 109	2
5	5.17	Cornoside	C_14_H_20_O_8_		315.1089	153, 123	2
6	5.26	Vanillic acid-hexoside	C_14_H_18_O_9_		329.0881	167, 152, 108	2
7	5.52	Glucovanillin	C_14_H_18_O_8_		313.0932	161	2
8	5.76	Chlorogenic acid	C_16_H_18_O_9_		353.0873	191, 179	1
9	5.94	Caffeoylquinic hexoside	C_22_H_28_O_14_		515.1408	191, 161	2
10	6.61	Coumaroylquinic acid	C_16_H_18_O_8_		337.0933	191, 163	2
11	6.98	Ionone-hexoside	C_19_H_28_O_11_	477.1614	431.1567	269, 161	3
12	7.81	Caffeoylquinic acid	C_16_H_18_O_9_	399.0931	353.0873	191	2
13	8.24	Caffeoylquinic acid	C_16_H_18_O_9_		353.0875	191, 170	2
14	8.38	Acylated hexose-pentose	C_18_H_24_O_11_	461.1301	415.125	121	3
15	8.76	Benzyl beta-primeveroside	C_19_H_28_O_12_	447.1507	401.1458	269, 161	2
16	9.4	Benzyl beta-primeveroside isomer	C_19_H_28_O_12_	447.1507	401.1458	269, 161	2
17	16.00	Quercetin O-hexose deoxyhexose	C_27_H_29_O_16_		609.1463	300, 301	2
18	16.29	Quercetin O-hexose deoxyhexose	C_27_H_29_O_16_		609.1463	300, 301	2
19	16.53	Quercetin O-hexose	C_21_H_20_O_12_		463.0885	300, 301	2
20	16.64	Quercetin O-pentose hexose	C_26_H_28_O_16_		595.1310	300, 301	2
21	17.08	Quercetin O-pentose	C_20_H_18_O_11_		433.0777	300, 301, 271	2
22	17.32	unknown	C_23_H_33_O_13_	517.1929		300, 301, 271	
23	17.79	Quercetin O-pentose	C_20_H_17_O_11_		433.0777	300, 301, 271	2
24	18.05	Quercetin O-hexose deoxyhexose	C_27_H_29_O_16_		609.1463	300, 301	2
25	18.21	Isorhamnetin O-hexose deoxyhexose	C_28_H_32_O_16_		623.1619	315, 300	2
26	18.27	Quercetin O-deoxyhexose	C_21_H_20_O_11_		447.0983	301, 300	2
27	19.46	Luteolin O-hexose deoxyhexose	C_27_H_30_O_15_		595.1519	285	2
28	22.16	Trihydroxy-octadecadienoic acid	C_18_H_32_O_5_		327.2183	229, 211	2
29	22.4	Trihydroxy-octadecenoic acid	C_18_H_34_O_5_		329.2338	229, 212	2

^a^ According to metabolomics standards initiative (MSI): level 1, unambiguous identification with reference standards; level 2, putative identification by matching MS2 data to the literature data or spectral databases; level 3, putative identification established by spectral similarity to chemical class of compounds and chemotaxonomic data.

**Table 3:** Experimental design matrix of the Box–Behnken design and corresponding experimental, RSM-predicted, and ANN-predicted values of total phenolic content (TPC), total flavonoid content (TFC), and DPPH radical scavenging activity.

Run	Factors			Experimental Values			RSM Predicted Values			ANN Predicted Values		
	X_1_	X_2_	X_3_	TPC	TFC	DPPH	TPC	TFC	DPPH	TPC	TFC	DPPH
1	1	60	60	11.47 ± 0.05	9.37 ± 0.05	58.51 ± 0.02	11.25	8.79	61.23	11.48	8.84	57.83
2	5	100	60	18.21 ± 0.17	15.56 ± 0.16	80.05 ± 0.01	18.54	15.76	79.30	18.96	15.30	79.95
3	5	100	20	19.26 ± 0.06	13.30 ± 0.20	71.76 ± 0.01	20.48	15.43	74.26	19.56	13.29	72.72
4	9	60	60	13.91 ± 0.15	11.21 ± 0.09	68.79 ± 0.01	14.38	10.59	69.32	14.74	11.23	70.54
5	5	60	40	13.98 ± 0.27	10.57 ± 0.18	57.04 ± 0.01	13.74	14.11	58.76	13.25	10.14	61.19
6	5	20	60	15.09 ± 0.06	7.27 ± 0.29	71.94 ± 0.02	14.49	7.16	69.45	15.78	7.91	76.85
7	1	60	20	9.46 ± 0.27	7.32 ± 0.12	65.96 ± 0.01	8.05	6.82	65.43	9.96	7.29	68.72
8	9	60	20	14.43 ± 0.03	13.27 ± 0.32	59.75 ± 0.01	14.64	13.86	57.03	14.96	13.29	59.72
9	1	20	40	9.28 ± 0.09	6.53 ± 0.19	78.10 ± 0.03	10.09	8.18	77.88	10.93	7.13	78.08
10	5	20	20	9.93 ± 0.13	7.18 ± 0.10	65.64 ± 0.02	9.60	8.78	66.40	8.69	7.69	66.34
11	9	20	40	11.19 ± 0.09	9.66 ± 0.07	67.33 ± 0.02	11.31	9.28	69.30	10.91	9.48	67.96
12	9	100	40	23.22 ± 0.16	21.65 ± 0.15	86.34 ± 0.01	22.42	20.22	86.57	23.64	21.21	86.75
13	1	100	40	14.03 ± 0.08	12.09 ± 0.04	80.27 ± 0.01	13.92	12.48	78.31	13.59	12.53	80.35
14	5	60	40	16.24 ± 0.25	14.18 ± 0.28	57.93 ± 0.02	13.74	14.11	58.76	16.66	14.89	58.47
15	5	60	40	14.18 ± 0.07	12.11 ± 0.12	61.30 ± 0.02	13.74	14.11	58.76	14.58	12.46	61.53

X_1_: Extraction time (min); X_2_: Ultrasound amplitude %; X_3_: Water concentration %; TPC: total phenolic content (mg GAE/g DW); TFC: total flavonoid content (mg CE/g DW); DPPH: 2,2-diphenyl-1-picrylhydrazyl radical scavenging activity (% inhibition); RSM: Response Surface Methodology; ANN: Artificial Neural Network. Experimental values are expressed as mean ± standard deviation (n = 3).

**Table 4:** Analysis of variance (ANOVA) for TPC, TFC, and DPPH responses.

Source	DF	Sum of Squares	Mean Square	F Ratio	*p*-Value
TPC					
RMSE					1.27
R^2^					0.96
Adj. R^2^					0.89
Model	9	201.62	22.40	13.96	0.0048
Error	5	8.02	1.60		
Total model	14	209.65			
Lack of Fit	3	4.90	1.63	1.05	0.5223
Pure error	2	3.12	1.56		
Total error	5	8.02			
TFC					
RMSE					1.52
R^2^					0.95
Adj. R^2^					0.85
Model	9	209.80	23.31	10.13	0.0101
Error	5	11.51	2.30		
Total model	14	221.32			
Lack of Fit	3	4.92	1.64	0.50	0.7205
Pure error	2	6.59	3.29		
Total error	5	11.51			
DPPH					
RMSE					3.06
R^2^					0.96
Adj. R^2^					0.89
Model	9	1144.76	127.20	13.58	0.0052
Error	5	46.85	9.37		
Total model	14	1191.61			
Lack of Fit	3	36.77	12.26	2.43	0.3047
Pure error	2	10.10	5.04		
Total error	5	46.85			

TPC: total phenolic content; TFC: total flavonoid content; DPPH: 2,2-diphenyl-1-picrylhydrazyl radical scavenging activity; DF: degrees of freedom; RMSE: root mean square error; R^2^: coefficient of determination; Adj. R^2^: adjusted coefficient of determination. Lack-of-fit was not significant (*p* > 0.05), indicating adequate model fit.

**Table 5:** Mathematical model coefficients and their statistical significance.

Source	Estimate	Std Error	t-Ratio	Prob > |t|
TPC				
Intercept	13.74	0.71	19.40	<0.0001 *
Linear				
X_1_	2.43	0.43	5.62	0.0025 *
X_2_	3.73	0.43	8.63	0.0003 *
X_3_	0.74	0.43	1.71	0.1484
Interaction				
X_1_X_2_	1.82	0.61	2.97	0.0311 *
X_1_X_3_	−0.87	0.61	−1.41	0.2165
X_2_X_3_	−1.71	0.61	−2.79	0.0384 *
Quadratic				
X_1_^2^	−1.50	0.64	−2.35	0.0652
X_2_^2^	2.20	0.64	3.45	0.0182 *
X_3_^2^	−0.16	0.64	−0.24	0.8167
TFC				
Intercept	14.11	0.59	23.79	<0.0001 *
Linear				
X_1_	2.21	0.36	6.09	0.0017 *
X_2_	3.81	0.36	10.50	0.0001 *
X_3_	−0.32	0.36	−0.89	0.4148
Interaction				
X_1_X_2_	1.66	0.51	3.24	0.0231 *
X_1_X_3_	−1.31	0.51	−2.55	0.0515
X_2_X_3_	0.49	0.51	0.95	0.3875
Quadratic				
X_1_^2^	−1.67	0.53	−3.12	0.0262 *
X_2_^2^	0.10	0.53	0.19	0.8603
X_3_^2^	−2.43	0.53	−4.54	0.0062 *
DPPH (RSA)				
Intercept	58.76	1.77	33.25	<0.0001 *
Linear				
X_1_	−0.08	1.08	−0.07	0.9456
X_2_	4.43	1.08	4.09	0.0094 *
X_3_	2.02	1.08	1.87	0.1206
Interaction				
X_1_X_2_	4.21	1.53	2.75	0.0402 *
X_1_X_3_	4.12	1.53	2.69	0.0431 *
X_2_X_3_	0.50	1.53	0.33	0.7578
Quadratic				
X_1_^2^	5.08	1.59	3.19	0.0243 *
X_2_^2^	14.17	1.59	8.90	0.0003 *
X_3_^2^	−0.59	1.59	−0.37	0.7283

X_1_: extraction time; X_2_: ultrasound amplitude; X_3_: water concentration in glycerin; TPC: total phenolic content; TFC: total flavonoid content; DPPH (RSA): DPPH radical scavenging activity. * indicates statistically significant coefficients (*p* < 0.05).

**Table 6:** Estimation parameters of the ANN model for TPC, TFC, and DPPH responses using training and validation datasets.

Parameter	TPC (Training)	TPC (Validation)	TFC (Training)	TFC (Validation)	DPPH (Training)	DPPH (Validation)
R^2^	0.95	0.99	0.93	0.98	0.98	0.85
RMSE	0.56	0.12	0.73	0.52	0.88	2.83
MSE	0.31	0.01	0.53	0.27	0.77	8.01
SSE	4.09	0.03	6.85	0.53	10.08	15.99
MAE	0.02		0.01		0.01	
MAPE (%)	1.79		1.39		1.64	

TPC: total phenolic content; TFC: total flavonoid content; DPPH: DPPH radical scavenging activity; R^2^: coefficient of determination; RMSE: root mean square error; MSE: mean square error; SSE: sum of squared errors; MAE: mean absolute error; MAPE: mean absolute percentage error.

**Table 7:** Student’s *t*-test analysis comparing glycerin and 70% ethanol for extraction efficiency.

	Group	Mean ± SD	t-Ratio	Difference	Prob ˃ |t|	Interpretation
TPC	Glycerin	21.16 ± 0.52 (mg GAE/g DW)	61.12315	11.66	<0.0001 *	Significant difference
	70% Ethanol	9.49 ± 0.21 (mg GAE/g DW)				
TFC	Glycerin	19.77 ± 0.45 (mg CE/g DW)	179.1117	1.93	<0.0001 *	Significant difference
	70% Ethanol	7.84 ± 0.02 (mg CE/g DW)				
DPPH RSA	Glycerin	81.03 ± 1.07 (%)	2.382807	2.16	<0.0381 *	Significant difference
	70% Ethanol	78.86 ± 0.82 (%)				

TPC: total phenolic content; TFC: total flavonoid content; DPPH RSA: DPPH radical scavenging activity; GAE: gallic acid equivalents; CE: catechin equivalents; DW: dry weight. Values are expressed as mean ± standard deviation (n = 3). * indicates statistically significant differences between solvents (*p* < 0.05) according to Student’s *t*-test.

## Data Availability

The original contributions presented in the study are included in the article, further inquiries can be directed to the corresponding author.

## Abbreviations

ANOVA	Analysis of Variance
ANN	Artificial Neural Network
BBD	Box–Behnken Design
CE	Catechin Equivalents
DAD	Diode Array Detector
DF	Degrees of Freedom
DPPH	2,2-Diphenyl-1-picrylhydrazyl
DW	Dry Weight
GAE	Gallic Acid Equivalents
MAE	Mean Absolute Error
MAPE	Mean Absolute Percentage Error
MSE	Mean Square Error
MS/MS	Tandem Mass Spectrometry
MSI	Metabolomics Standards Initiative
RMSE	Root Mean Square Error
RSA	Radical Scavenging Activity
RSM	Response Surface Methodology
SSE	Sum of Squared Errors
TFC	Total Flavonoid Content
TPC	Total Phenolic Content
UAE	Ultrasound-Assisted Extraction
UHPLC–HRMS	Ultra-High-Performance Liquid Chromatography–High-Resolution Mass Spectrometry
UV–Vis	Ultraviolet–Visible Spectrophotometer
